# Follow-Up of Patients with Multidrug Resistant Tuberculosis Four Years after Standardized First-Line Drug Treatment

**DOI:** 10.1371/journal.pone.0010799

**Published:** 2010-05-24

**Authors:** Guang Xue He, Yan Guang Xie, Li Xia Wang, Martien W. Borgdorff, Marieke J. van der Werf, Ji Huan Fan, Xing Lu Yan, Fa Bin Li, Xue Zhi Zhang, Yan Lin Zhao, Susan van den Hof

**Affiliations:** 1 National Center for Tuberculosis Control and Prevention, China Center for Disease Control and Prevention (China CDC), Beijing, China; 2 Center for Infection and Immunity Amsterdam (CINIMA), Academic Medical Center, University of Amsterdam, Amsterdam, The Netherlands; 3 Heilongjiang Provincial Center for Tuberculosis Control and Prevention, Harbin, Heilongjiang, China; 4 KNCV Tuberculosis Foundation, The Hague, The Netherlands; 5 Public Health School, Peking Union Medical College, Beijing, China; 6 National Tuberculosis Reference Laboratory, China Center for Disease Control and Prevention (China CDC), Beijing, China; Institute of Infectious Diseases and Molecular Medicine, South Africa

## Abstract

**Background:**

In 2004, an anti-tuberculosis (TB) drug resistance survey in Heilongjiang province, China, enrolled 1574 (79%) new and 421 (21%) retreatment patients. Multi-drug resistant (MDR) TB was detected in 7.2% of new and 30.4% of retreatment patients. All received treatment with standardized first-line drug (FLD) regimens.

**Methodology/Principal Findings:**

We report treatment outcomes of the 2004 cohort, and long-term outcomes as assessed in the second half of 2008. The reported cure rate for MDR-TB patients was 83% (94/113) among new and 66% (85/128) among retreatment patients (P<0.001). Ten of the 241 MDR-TB patients died during treatment. Of the remaining 231, 129 (56%) could be traced in 2008. The overall recurrence rates among new and retreatment cases were 46% and 66%, respectively (P = 0.03). The overall death rates among new and retreatment cases were 25% and 46%, respectively (P = 0.02). Forty percent of the traced new cases and 24% of the retreatment cases were alive and without recurrent TB (P = 0.01). Of the 16 patients who failed or defaulted from treatment in 2004, only two patients were not re-diagnosed with TB by 2008. Of the 111 (86%) patients with an initial successful treatment outcome 63 (57%) had developed recurrent TB, 40 (36%) had died, 27 (24%) of them died of TB. The follow-up period of four years precluded follow-up of all patients. In a highly conservative sensitivity analysis in which we assumed that all non-included patients were alive and did not have recurrent TB, the recurrence and death rate were 33% and 21%.

**Conclusions/Significance:**

Documentation of cure based on conventional smear microscopy was a poor predictor of long term outcomes. MDR-TB patients in Heilongjiang province in China had high recurrence and death rates four years after treatment with standardized FLD regimens, reinforcing the need for early diagnosis and treatment of MDR-TB, including assessment of treatment outcomes with more sensitive laboratory methods.

## Introduction

With over 9 million cases and nearly 2 million deaths annually, tuberculosis (TB) remains a major cause of morbidity and mortality worldwide [1]. One of the important challenges for TB control is drug resistance, particularly multidrug resistant (MDR) and extensively drug-resistant (XDR) TB [2], [3]. MDR-TB is defined as resistance of *Mycobacterium tuberculosis* isolates to the two most effective first-line drugs (FLD), i.e., rifampicin and isoniazid, while XDR-TB has additional resistance to any fluoroquinolone and at least one of the three injectable second-line anti-TB drugs (i.e. amikacin, kanamycin, capreomycin) [4], [5]. MDR-TB and XDR-TB treatment relies on regimens with less effective and more toxic second-line drugs (SLD). Failure rates of MDR-TB patients treated with standard World Health Organization (WHO) FLD treatment regimens ranged 4%–47% among new cases and 21%–50% among retreatment cases [6].

China has the second largest number of TB cases in the world [1], and has a high prevalence of drug resistant TB [7]. The first national drug resistance survey (DRS) in 2007 reported an overall MDR-TB prevalence of 8.3%, of which 8% were XDR-TB [8]. Some TB hospitals in China have also reported TB strains that are resistant to all SLD [9], [10].

Heilongjiang province is located in the northeast of China and has a population of 38.1 million. From 2004–2008, the case notification rate for all TB was 90–107/100 000 and, for smear-positive TB, it was 50–57/100 000. The WHO recommended TB control strategy, known as DOTS, was implemented progressively in this province starting in 1992, and DOTS coverage reached 100% in 1995. In 2004, Heilongjiang province joined the global project on anti-tuberculosis drug resistance surveillance organized by the World Health Organization and the International Union Against Tuberculosis and Lung Disease (WHO/IUATLD). In this survey, Heilongjiang province contributed 1995 isolates, including 1574 (78.9%) from new TB cases. The prevalence of any drug resistance in this survey was 36.1% among new cases and 67.5% among retreatment cases, i.e., TB patients who received at least one month of anti-TB treatment in the past. The MDR prevalence was 7.2% among new patients and 30.4% among retreatment patients. [11].

Up to 50% of MDR-TB patients treated with standardized first-line DOTS regimens seem to be treated successfully at the end of the treatment period [6], [12]–[14]. In China, the cure rate of MDR-TB patients at the end of FLD treatment was relatively high based on sputum smear microscopy results [15]–[17]. Whether long term outcomes in China are also good is unknown, since there is a paucity of data on long term efficacy of DOTS regimens for treatment of drug-resistant TB, especially under routine program conditions [18], [19]. To fully evaluate the treatment outcome of MDR-TB patients, longer follow-up is needed. We report on treatment outcomes of patients included in a representative drug resistance survey in Heilongjiang province in China, and on long term health status of MDR-TB patients as assessed four years after the end of standardized first-line treatment.

## Methods

### Ethics statement

The research project was approved by the Chinese Ethical Committee for TB Operational Research. Written informed consent was obtained from individuals before an interview was conducted and sputum samples were collected. If the individual had died, written informed consent was obtained from the family member before they were interviewed. Patients and TB hospitals were informed of the sputum smear, culture, and DST test results, and MDR-TB patients identified during follow-up were requested to go to the TB hospital for treatment, including with second-line drugs.

### Drug resistance survey in 2004

For the drug resistance survey (DRS), the “Guidelines for surveillance of drug resistance in tuberculosis” developed by WHO/IUATLD were followed [20]. Methods, including those for quality control of drug susceptibility testing (DST), have been described in more detail previously [11]. In Heilongjiang province, 30 out of 113 counties and districts were randomly selected, and all smear-positive cases diagnosed in these sites during the study period (January through December 2004) were eligible for inclusion [21]. DST was done for FLDs: isoniazid (H), rifampicin (R), streptomycin (S), and ethambutol (E).

### Treatment regimens and outcomes

As no established program for treating drug-resistant TB existed in China in 2004, Heilongjiang province followed the National Tuberculosis Programme (NTP) treatment guidelines for all patients. Treatment of new smear-positive patients consisted of 2 months of isoniazid, rifampicin, pyrazinamide, and ethambutol followed by 4 months of isoniazid and rifampicin three times weekly (2H_3_R_3_Z_3_E_3_/4H_3_R_3_). Patients who had previously received at least one month of TB treatment (i.e. retreatment patients) received 2 months of isoniazid, rifampicin, pyrazinamide, streptomycin and ethambutol, followed by 6 months of isoniazid, rifampicin, and ethambutol three times weekly (2H_3_R_3_Z_3_E_3_S_3_/6H_3_R_3_E_3_.). Drug intake was observed at the health facility throughout treatment.

Treatment outcomes were assessed using international definitions and were recorded in routine treatment registers [22]. Treatment success is defined as either cure or treatment completed. Cure is defined as a patient who is sputum smear-negative in the last month of treatment and on at least one previous occasion. Treatment completed is defined as a patient who has completed treatment but who does not meet the criteria to be classified as a cure or a failure. Treatment failure is defined as a patient who is sputum smear-positive at 5 months or later during treatment. Default is defined as a patient whose treatment was interrupted for two consecutive months or more. Transfer out is defined as a patient who has been transferred to another recording and reporting unit and whose treatment outcome is unknown. Death is defined as a patient who dies for any reason during the course of treatment. The cause of death during TB treatment was not recorded.

### Follow up of MDR-TB patients in 2008

First, all information in the original questionnaires for all patients included in the DRS was re-checked against medical files, treatment registers and DRS files. This information included demographics, treatment history, DST results, and treatment outcome. Re-checking identified very few discrepancies. Where discrepancies occurred, we used the information from county/district TB centers.

Second, staff from TB centers in the counties/districts included in the DRS were instructed on how to follow-up the 241 patients that were included in the 2004 DRS and had MDR-TB. County/district level staff and one provincial staff member interviewed patients in their home in the period June–December 2008. During the interview, participants were asked about presence of common TB symptoms (i.e., cough, haemoptysis, chest pain, fever, fatigue, anorexia and night sweat), and whether they had been diagnosed with (recurrent) TB since 2004. Three sputum samples were collected from all participants for microscopy, culture and DST using the same methods as in the 2004 survey. If the MDR-TB patient had been diagnosed with recurrent TB after the end of treatment, the patient was asked for the name of the hospital or TB center where the recurrence was diagnosed and the date of diagnosis. Staff rechecked the smear examination results, diagnosis and dates with the medical files in the facility. If the MDR-TB patient had died, family members were interviewed about the time of death and its cause, and staff would recheck the cause with the death certificate provided by the hospital. Patients who had TB registered as a cause of death were assumed to have had recurrent TB. Patients who had moved were not traced. A random ten percent of the interviews were repeated by provincial staff not involved in the initial interview. The re-interviewing results gave almost identical results except for a few dates.

### Data management and analysis

Data were double entered in EpiData 3.1 (The EpiData Association, Odense, Denmark, 2003–2008) and discrepancies were checked against the raw data. Analysis was performed using SPSS 13.0 (SPSS Inc. Chicago, IL, USA). Differences between groups in the distribution of categorical variables were tested with the chi-quare test. For the analysis of treatment outcome from initial TB treatment, we compared patients who were cured to those that failed, died, or defaulted in a multivariate logistic regression model. Variables available were living area, sex, age, drug resistance pattern, bacillary load, and number of treatment episodes. Adjusted odds ratios (OR) and 95% confidence intervals (CI) were assessed by backward selection of variables included in the multivariate model, based on the fit of the model as tested with the likelihood ratio chi-square test (p = 0.05).

## Results

### Outcomes after initial TB treatment

**Figure 1:**
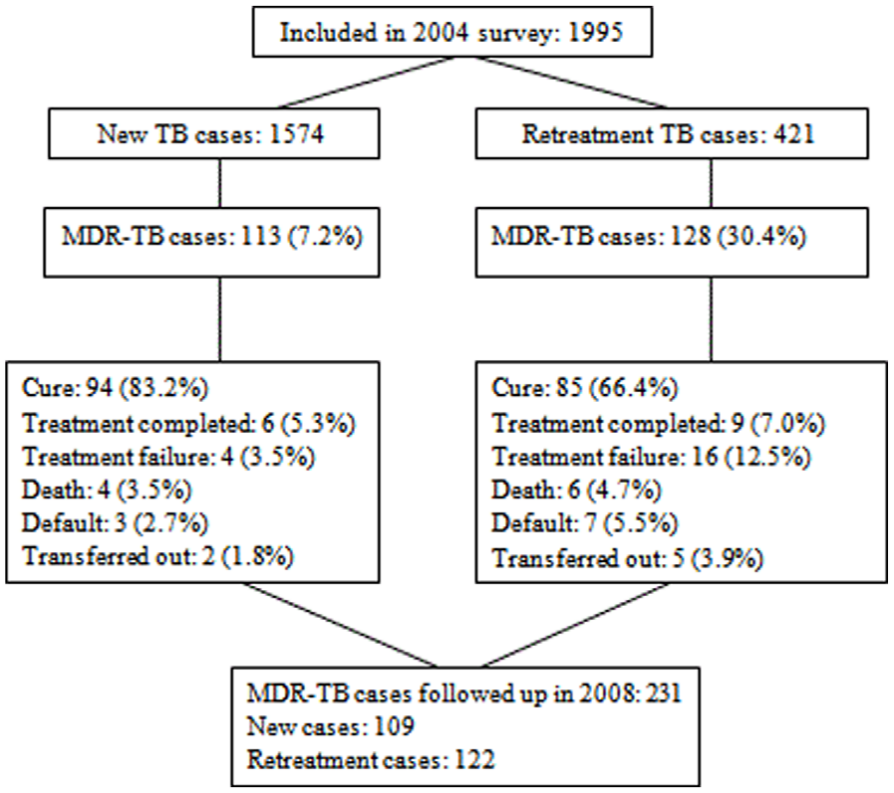
Flow chart on numbers of patients included in the anti-tuberculosis drug resistance surveillance in 2004 in Heilongjiang province, China, and followed up in 2008.

Initial treatment outcome data were available for all 1574 (79%) new smear-positive TB patients and 421 (21%) retreatment smear-positive TB patients included in the 2004 survey. The reported cure rate was 89% (1405/1574) among new and 74% (311/421) among retreatment patients (P<0.001). The reported cure rate for MDR-TB patients was 83% (94/113) among new and 66% (85/128) among retreatment patients (P<0.001) (Figure 1).

Among new patients, those with a sputum smear grade 4+ at diagnosis (OR = 0.6, 95% CI 0.4–0.9) and multidrug resistance (OR = 0.5, 95% CI 0.3–0.9) were less likely to be cured. Among retreatment patients, those living in a city (OR = 0.5, 95% CI 0.3–0.8), with at least two previous TB treatment episodes (OR = 0.5, 95% CI 0.3–0.8), and with multidrug resistance (OR = 0.5, 95% CI 0.3–0.8) were less likely to be cured.

### Follow up of MDR-TB patients

Among 241 new and retreatment patients with MDR-TB, 10 (4%) died during treatment. We attempted to trace the remaining 231 patients. We were able to interview 129 (56%) patients or their household members. The average duration between the end of treatment and follow up was 47 months (range 42–54) for new cases and 45 months (range 41–52) for retreatment cases. Reasons for non-inclusion were: having transferred out (n = 46, 19%), working somewhere else as a migrant worker (n = 28, 12%), lost (n = 21, 9%), and refusal to participate (n = 7, 3%) (Table 1).

**Table 1:** Treatment outcome of 241 multidrug resistant tuberculosis patients diagnosed and treated in 2004 and then followed up in 2008*.

Status at follow-up in 2008	Initial treatment outcome from TB episode in 2004						
	Cure	TC	Failure	Death	Default	TO	Total (%)
No recurrence	46	2	0	0	2	0	50 (38.8)
*- death, not due to TB*	*12*	*1*	*0*	*0*	*1*	*0*	*14 (5.8)*
Recurrence	55	8	9	0	5	2	79 (61.2)
*- SS+ and/or C+ in 2008*	*22* **	*1* **	*5*	*0*	*2*	*0*	*30 (23.3)* ***
*- death due to TB*	*22*	*5*	*4*	*0*	*3*	*2*	*36 (27.9)*
Subtotal with follow-up	101	10	9	0	7	2	129 (53.5)
Transferred out	28	3	9	0	2	4	46 (19.1)
Migrant workers	24	1	1	0	1	1	28 (11.6)
Refused	6	1	0	0	0	0	7 (2.9)
Lost	20	0	1	0	0	0	21 (8.7)
Died during treatment in 2004	0	0	0	10	0	0	10 (4.2)
Total	179	15	20	10	10	7	241 (100.0)

TC = Treatment completed; TO = Transferred out; SS+ = sputum smear positive; C+ = culture positive.

*The data include 113 new and 128 retreatment MDR-TB cases.

**SS+C+: 19; SS−C+: 4.

***SS+C+: 22; SS+C−: 2; SS−C+: 6.

Among the 129 cases traced, 79 (61%) were again diagnosed with TB after 2004. Of these 79 patients with recurrent TB, 36 (46%) died of TB and 30 (38%) were found to be sputum smear or culture positive at the time of interview. Of the 16 patients who failed or defaulted from treatment in 2004, only two patients were not re-diagnosed with TB (Table 1).

Of the 129 traced MDR-TB patients, 111 (86%) were recorded in the TB register as having been successfully treated after diagnosis in 2004 (Table 1). Of those 111, 63 (57%) had a documented recurrence of TB. Most of the 63 patients with recurrent TB either died from TB (27; 43%) or were sputum smear or culture positive at the time of the interview (23; 37%). The recurrence rate among those treated successfully in 2004 increased year by year, and the overall recurrence rate among retreatment cases was significantly greater than the recurrence rate among new cases (66% vs. 46%, P = 0.03). The overall death rate and death rate due to TB was also greater among retreatment cases than new cases (46% vs. 25%, P = 0.02; 36% vs. 12%, P = 0.003) (Table 2).

**Table 2:** Timing of TB recurrence and death for 111 multi-drug resistant tuberculosis patients evaluated four years after being successfully treated for TB in 2004.

Timing of TB recurrence or death, in months after end of treatment	New cases (n = 52)								Retreatment cases (n = 59)							
	TB recurrence				Death				TB recurrence				Death			
	n	cum. n (cum. %)	SS/C+	cum. n ( cum. %)	n	cum. n (cum. %)	n due to TB	cum. n (cum. %)	n	cum. n (cum. %)	SS/C+	cum. n (cum. %)	n	cum. n (cum. %)	n due to TB	cum. n (cum. %)
≤12	5	5 (9.6)	3	3 (5.8)	2	2 (3.8)	0	0 (0.0)	12	12 (20.3)	2	2 (3.4)	9	9 (15.3)	7	7 (11.9)
13–24	6	11 (21.2)	1	(4 (7.7)	5	7 (13.5)	2	2 (3.8)	10	22 (37.3)	4	6 (6.8)	8	17 (28.8)	6	13 (22.0)
25–36	7	18 (34.6)	1	5 (9.6)	3	10 (19.2)	3	5 (9.6)	10	32 (54.2)	2	8 (13.6)	7	24 (40.7)	6	19 (32.2)
37+	6	24 (46.2)	5	10 (19.2)	3	13 (25.0)	1	6 (11.5)	7	39 (66.1)	5	13 (22.0)	3	27 (45.8)	2	21 (35.6)

cum. = cumulative.

SS/C+ = Sputum smear or culture was positive at time of follow-up.

Comparison of recurrence rate between new cases and retreatment cases: Pearson X^2^ = 4.481, P = 0.03.

Comparison of SS/C+ TB recurrence rate between new cases and retreatment cases: Pearson X^2^ = 0.132, P = 0.72.

Comparison of death rate between new cases and retreatment cases: Pearson X^2^ = 5.169, P = 0.02.

Comparison of death due to TB between new cases and retreatment cases: Pearson X^2^ = 8.318, P = 0.003.

It is possible that recurrence and death rates were lower in patients that could not be traced. If we assume that all patients who were not followed-up were alive and did not have recurrent TB, the recurrence rate was 33% (63/194) and the death rate was 21% (40/194). If we assume that only those who were not followed-up because they were migrant workers were alive and did not have recurrent TB, the recurrence rate and death rates were 46% (63/136) and 29% (40/136), respectively (Table 3).

**Table 3:** Sensitivity analysis of recurrence and death in 2008 among multidrug resistant tuberculosis patients who were treated successfully after diagnosis in 2004.

	Recurrence		Death	
	n*/N	%	n/N	%
All interviewed cases	63/111	56.8	40/111	36.0
Assuming that migrant workers did not have recurrent TB	63/136	46.3	40/136	29.4
Assuming that all non-interviewed cases did not have recurrent TB	63/194	32.5	40/194	20.6

*including 27 cases who died of TB.

## Discussion

In this large study of patients with MDR-TB in China, we found that patients with MDR-TB who had been declared cured with first-line anti-TB treatment had a high rate of TB recurrence and death within 4 years.

As observed in other studies [13], MDR-TB was associated with an increased risk of failure after standardized first-line drug treatment. The reported cure rates in Heilongjiang of 83% for new cases and 66% for retreatment cases are based on smear microscopy and are high compared to what is observed in other countries [6], [13], [14], [18], but similar to reports from other Chinese provinces [15]–[17]. A possible explanation for the reported high cure rate of MDR-TB in China might be insufficient sensitivity of sputum smear examination. Although quality assurance for smear microscopy has been implemented in China for several years, the impact of such a program in this Chinese province may have been inadequate or the sensitivity of microscopy for determining treatment outcome, regardless of adequate quality assurance, may be insufficient for patients with (MDR-)TB. Patients with MDR-TB who receive FLD treatment may have their bacillary burden lowered just enough to avoid detection by light microscopy. Newer technologies may be needed. For example, light-emitting diode (LED) – based fluorescence microscopy (FM) offers increased sensitivity for the evaluation of sputum smear samples for TB compared with conventional light microscopy [23]. Improving classification of TB patients at the end of treatment may require more vigorous implementation of smear microscopy quality assurance, implementation of new tools (e.g., LED-FM), or implementation of existing but underutilized, tools (e.g., culture).

Our study reports the largest number of MDR-TB patients with long-term follow-up after initial treatment with FLD. We found that the rate of TB recurrence and death was high four years after MDR-TB patients were judged to have been cured, regardless of assumptions that we made in our analysis. Including only those patients with follow-up data, we found that the rate of recurrence was 56% and death 36%; making the highly conservative assumption that all non-included patients were alive and did not have recurrent TB, the recurrence rate and death rate were 33% and 21%, respectively.

In the first year after treatment, 15% of the MDR-TB patients that were successfully treated had a recurrence of TB and 10% died. Both the TB recurrence and death rates in new cases diagnosed with MDR were lower than in retreatment cases diagnosed with MDR. It is possible that retreatment patients had more extensive drug resistance at the time of their 2004 treatment episode, making them less likely to have any bacillary load reduction from FLD treatment.

Our study has important implications for TB control in China. High rates of recurrence and death impact the patient and, even more important, increase the number of persons in the community with infectious MDR-TB. As would be expected treatment with FLD is highly ineffective in curing MDR-TB even if the reported cure rate is high. In regions with a high prevalence of drug-resistance, such as this province in China, drug-susceptibility testing will need to be scaled up. At a minimum, such testing should be performed in all patients at high risk of MDR-TB, such as known contacts of MDR-TB patients and re-treatment patients, and may need to be rapidly scaled up to all patients, depending on the community prevalence of MDR-TB and available resources.

There are several limitations to our study. The main limitation is that only slightly more than half of patients could be included in the follow-up study, which may have led to an overestimation of recurrence and death rates. A highly conservative sensitivity analysis in which we assumed that all the non-included patients were alive and did not have recurrent TB, still showed high recurrence and death rates. This conservative estimate can be assumed to be an underestimate of recurrence given the TB incidence among migrant populations [23], [24]. It is possible that we under-estimated TB-related deaths by only counting as TB-related deaths those that had TB recorded on their death certificates. We were also not able to differentiate whether recurrent TB was due to relapse or re-infection, because we did not perform genotyping of TB strains. However, in the first year, 15% had recurrent TB which is very high and most likely due to relapse. Unfortunately, due to limited resources, non-MDR-TB patients were not followed up. Therefore, we could not compare long-term outcomes of MDR-TB patients with the long term outcomes of non MDR-TB patients. In Beijing, 272 sputum smear positive pulmonary TB patients treated successfully were followed up for 5 years after end of treatment. The recurrence and death rates were 3.7% and 9.4%, respectively, which is much lower than in our study [25]. This may be due to the much lower prevalence of drug resistance [26], as also indicated by a low failure rate of 1.6%. In China as well as in Heilongjiang province, recurrent TB accounts for 9.0% of all notified smear positive TB cases [1]. In a recent review, a large degree of variation was found in tuberculosis recurrence in non MDR-TB patients ranging from 2.8% to 12.3% with WHO recommended DOTS after successful treatment [27]. A more recent large case-control study from Hong Kong found intermittent (three times weekly) treatment to be significantly associated with recurrence when compared with daily treatment [28]. In China, intermittent regimens are used, which may contribute to higher recurrence rates.

In a few pilot areas in China, treatment of drug-resistant TB is now being implemented through the national TB program. WHO guidelines recommend treatment with at least 4 drugs with certain or almost certain effectiveness [29]. Only in these pilot sites and a few well-developed areas of China is DST performed routinely for MDR-TB suspects and results used to guide TB treatment. Based on our study results, we strongly suggest that China rapidly scale-up access to MDR-TB diagnosis and treatment around the country, with priority for provinces with high anti-TB drug resistance prevalence.

In conclusion, MDR-TB patients in Heilongjiang province in China had high recurrence and death rates four years after treatment with standardized FLD regimens. Documentation of cure based on conventional smear microscopy at the end of treatment was a poor predictor of long-term outcome. Adequate identification and treatment of MDR-TB patients is urgently needed in order to improve long-term outcomes and to control the spread of MDR-TB.
